# Oral Health Attitudes among Preclinical and Clinical Dental Students in Germany

**DOI:** 10.3390/ijerph17124253

**Published:** 2020-06-15

**Authors:** Mohamed Mekhemar, Jonas Conrad, Sameh Attia, Christof Dörfer

**Affiliations:** 1 Clinic for Conservative Dentistry and Periodontology, School of Dental Medicine, Christian Albrechts-University of Kiel, Arnold-Heller-Str. 3, Haus B, 24105 Kiel, Germany; conrad@konspar.uni-kiel.de (J.C.); doerfer@konspar.uni-kiel.de (C.D.); 2 Department of Cranio Maxillofacial Surgery, Justus-Liebig University Giessen, Germany, Klinik Str. 33, 35392 Giessen, Germany; sameh.attia@dentist.med.uni-giessen.de

**Keywords:** oral health attitudes, dental students, Germany, oral hygiene

## Abstract

Oral health care providers are expected to show good examples of oral health behaviours and attitudes to their community. Previous studies displayed the constructive effect of dental education on oral hygiene manners of undergraduate students. The aim of this survey was to assess and compare aspects of oral health attitudes and behaviours between preclinical and clinical dental students in German universities. The German-language version of the HU-DBI was distributed to preclinical and clinical students from different German universities. Dichotomized (agree/disagree) responses to 20 HU-DBI items were provided in this study, with a maximum possible score of 19. A quantitative estimate of oral health attitudes and behaviours was provided by the total of appropriate answers given to every statement by each group. Data were analysed statistically. The overall mean score of answers favouring good oral hygiene was marginally higher in preclinical (14.62) than clinical students (14.31) but showed no statistical significance. Similarly, the analysis of each item individually displayed no statistically significant differences between preclinical and clinical participants, except in a single item of the survey. This study showed no effective differences in oral hygiene attitudes and behaviour between preclinical and clinical students in German universities. This reveals a weak effect of dental education on improving students’ oral health attitudes in Germany and might demand the introduction of more courses emphasizing the importance of correct oral health behaviour of health care providers.

## 1. Introduction

Oral diseases are among the most prevalent diseases worldwide and produce abundant health and economic burdens, reducing quality of life for individuals and societies affected [1]. Health education is considered one of the fundamental elements in the success of disease prevention in several areas of health care, including oral and dental health [2]. Among the most reliable methods of oral health education, the attitudes and behaviour of oral health providers towards their own dental and oral hygiene can have a major impact on improving public oral health [3]. As oral health attitudes display their mind’s predisposition to oral health and reflect practically as oral health behaviour, oral health providers can advise patients and present good examples of correct oral practices to raise the awareness on oral disease prevention as one of their substantial responsibilities [4].

Historically, Germany has a long tradition of medical and dental excellence reaching back as far as the nineteenth century. In the late 19th and early 20th centuries, Germany was considered a peak of medical and dental education throughout the world [5]. To date, dental education in the Federal Republic of Germany is offered through 30 dental faculties of public or private universities in different German states [6]. Students must complete a regular course of study for ten semesters with an additional six months (11th semester). Succeeding a five-semester pre-clinical period, focusing on natural sciences, dental materials and general medicine, the student must successfully complete the preliminary dental examination. During this stage, students receive theoretical education about oral health care with minor practical application within the dummy-head training courses. This is followed by another five semesters of clinical study, including lectures, seminars and practical training. Finally, a degree in dentistry is awarded after successful completion of the second state-administered dentistry exam. This educational process includes a series of progressive stages that commence with theory and simulation and end with a clinical training phase in which the student must fulfil the tasks of a professional dentist. This sharp preclinical–clinical transition is of great educational importance for students, as their role shifts from being taught about oral hygiene and patient care to being responsible for real patients’ oral health [7]. Research highlights the importance of adjusting the dental curricula towards the strengthening of students’ professional competence during this transitional phase [7], allowing clinical students to integrate theory and practice and to show correct attitudes towards their own oral health, as well as a professional and healthy oral care behaviour as role models and educators to their patients [8,9].

Recently, several studies evaluated this educational transition in different regions of the world comparing the oral health attitudes and behaviour among preclinical and clinical dental students as primary signs for educational progress. Several outcomes displayed significant improvements of oral health manners in clinical students [4,10,11,12,13,14,15,16,17], whereas others observed a less effective preclinical–clinical transition [18,19]. Examining the literature, no similar investigation was found in Germany. Thus, this study aimed to assess the self-reported oral health behaviour and attitudes among preclinical and clinical dental students in Germany by using a modified Hiroshima University-Dental Behaviour Inventory (HU-DBI) questionnaire to provide an insight into the students’ educational transition to the clinical phase and its effect on their preparation as oral health educators. 

## 2. Materials and Methods

### 2.1. Study Population and Methodology

The Hiroshima University-Dental Behavioural Inventory (HU-DBI) was established by Kawamura to examine dental health behaviour, perceptions and attitudes. It has already been translated from Japanese into German and multiple other languages for cross-cultural comparisons [17,20,21]. Results of these translated versions, including the German translation, have been reported previously and exhibited good validity and reliability [20]. In the current study, data on oral health attitude and behaviour was collected using a shortened version of the HU-DBI questionnaire [3,22,23] consisting of 20 items describing aspects of oral health behaviour and attitudes. Each item consisted of a dichotomous response (Agree/Disagree) (Table 1). The questionnaire was transformed into an online-survey using a web-based survey tool (Unipark, QuestBack GmbH, Cologne). After the approval by the ethics committee of the University of Kiel (AZ D 431/17), the survey-link was sent by email to all registered dental students at the Federal Association of Dental Students in Germany (Bundesverband der Zahnmedizinstudierenden in Deutschland) (N = 90) and the University of Kiel (N = 117). A link to the survey was additionally posted on several social media platforms (e.g., Facebook) in groups related to dental students and education in Germany to increase the number of participants. Eligible participants were dental students attending courses of all semesters (Table 2). No exclusion criteria were defined (e.g., age, gender or nationality).

The introductory text in the survey briefly explained the research project and assured anonymity and voluntary participation. No financial incentives or gifts were given, and no personal information was gathered from the participants except their gender, university and academic semester.

### 2.2. Sample Size Calculation

To determine the number of responding students needed for a significant database, the following conditions were defined for the sample size calculation:

1. Number of sent emails to registered students (*N* = 207);
2. A confidence level of 95%;
3. A margin of error of 5%.

Based on these conditions it was determined that within the study population, at least 135 students were needed for a statistically significant sample size.

### 2.3. Hiroshima University-Dental Behavioural Inventory Score and Statistical Analysis

The HU-DBI provides a quantitative estimate of participants’ attitudes toward oral health based on the sum of agree/disagree responses [11,18]. The general oral health attitudes and behaviour of the students was evaluated by calculating a sum score of their dichotomous responses (Agree or Disagree) to the items of the survey as described previously [11,18] by giving one point to each answer in favour of good oral health (Table 1). In the current investigation with 20 survey items, the maximum possible score was 19 points as the first item addressed sociodemographic information. Higher scores represented better oral health attitudes and behaviour.

The Shapiro–Wilk-Test was performed to test for normality of the data. Data was not normally distributed. Differences between preclinical and clinical semesters, as well as male students and female students’ sum scores were assessed by the Wilcoxon signed-rank test. In addition, every item was analysed individually by the chi-square test comparing the “Agree” responses of preclinical and clinical students to this item of the survey [24]. Data analysis was performed by SPSS software (SPSS Inc., Version 18.0.0, Chicago, IL, USA). The significance level (*p*-Value) for all tests was set at 0.05.

## 3. Results

A total of 172 dental students participated in the survey resulting in a statistically significant sample size and a response rate of 83%. Participants included 56 (32%) preclinical (semesters 1–5) and 116 (67%) clinical (semesters 6–11) students. Approximately 69% of the students were females and 31% males (Table 2). Around one third of the participating students were registered at the University of Kiel, while the remaining participants were from other German universities (Table 3). Less than a quarter (14–15%) of both student groups were still living with their families during their university studies. The percentage distribution of participants’ responses is presented in (Table 4).

### 3.1. Oral Health Attitudes 

Both groups showed a near 100% rate of previous visits to the dentist. Nevertheless, preclinical dental students showed better oral health attitudes than the clinical group in most of the items related to oral health attitudes. The number of clinical students who believed that brushing teeth was possible without toothpaste (9.48%) was significantly higher than preclinical students (1.79%) (*p* < 0.05) (Table 4).

### 3.2. Oral health Behaviour and Self-Reported Oral Health

The clinical students showed higher frequencies of toothbrushing, at least twice a day (92.24%) and after every meal (6.90%), than preclinical participants (87.50% and 1.79%, respectively). Nevertheless, in most of the items related to oral health behaviour and self-reported oral health, clinical students presented similar or inferior results to the preclinical group (Table 4).

### 3.3. Mean HU-DBI Scores and Statistical Significance

The overall HU-DBI mean score of answers favouring a good oral hygiene was marginally higher in preclinical (14.62 ± 0.42) than clinical students (14.31 ± 0.43) with no statistical significance (*p* = 0.170). Similarly, the analysis of each item of the survey displayed no statistically significant differences between preclinical and clinical participants, except in item 14 of the survey (*p* = 0.05) (Table 4). Comparing both genders of the participants, females displayed a significantly higher HU-DBI score (14.64 ± 0.42) than males (13.33 ± 0.44) (*p* = 0.001) (Table 5).

## 4. Discussion

In Germany, similarly to other European and non-European developed nations, oral diseases are considered among the most widespread diseases affecting the public health [25]. In recent years, significant oral health developments have been introduced to the German oral health system leading to decreased rates of oral disease detected in the population [25]. Nevertheless, up to the present time, no investigation assessed the oral health attitudes and behaviour of dental students in Germany as future oral health professionals. Owing to this deficiency, this study aimed to assess and compare aspects of oral health attitudes and behaviour between preclinical and clinical dental students in German universities to evaluate the didactic preclinical–clinical transition and the preparedness of clinical dental students as future dentists of the society and health educators to their patients.

In accordance with the common gender distribution in dental education [26], the current study population comprised 69% females and 31% males. Participation rates from students of the University of Kiel were higher (33%) than other universities as they were directly contacted by the University register. Only nearby 14% of the study participants resided with their families during their university semesters, as similarly described in other countries of northern Europe (Item 1) [27]. Among all students, females showed better oral health care than males as reported previously [17,18,28,29]. This difference was similarly observed in the general German population [30] and could be attributed to the fact that women usually care more about their appearance and body and thus may be more concerned about accepting behaviours and habits that promote their dental health [29]. The results revealed that the majority of dental preclinical and clinical students of the study population care about their oral health. Nevertheless, the more frequent regular dental visits among preclinical dental students exhibited contrasting results to similar studies in other countries as Turkey [4], Egypt [10] and Saudi Arabia [11] (Item 3). This might be due to the possibility of regular dental check-ups of clinical students during their clinical semester courses by their fellow students or supervisors. However, the overall rate of regular dental visits of both groups showed the highest percentage compared to similar studies performed in the Middle East, Asia and Europe [4,10,11,31].

In accordance with previous studies from Turkey, Jordan, Egypt, Saudi Arabia, Lithuania and India [4,10,11,32], clinical students in Germany showed more frequent toothbrushing at least twice daily or after every meal (Items 4 and 5) than their preclinical fellow students. However, in strong discrepancy with previous investigations [4,10,11,31], preclinical dental students in Germany indicated being more attentive about their dentition and oral health and caring more about their dental aesthetics and halitosis (Items 13 and 16). Furthermore, preclinical participants using a professional brushing technique (Item 7) for each of their teeth (Item 9), as well as a more frequent use of dental floss (Item 11) and mouthwashes (Item 12) unexpectedly demonstrated higher percentages. Moreover, clinical students correspondingly stated being bothered about their gingival aesthetics (Item 15), using soft tissue-unfavourable tooth brushes (Item 8) [33] and reported bleeding gums (Item 6) more than the preclinical group, indicating better self-reported periodontal health of the preclinical students. Clinical dental students were also significantly more likely to clean their teeth without toothpaste (Item 14) compared to preclinical participants, in compliance with previous investigations in Turkey [4], Egypt [10] and India [34]. In addition, most of the preclinical students thought that their teeth would get worse despite daily brushing (Item 10), reflecting less oral health knowledge as described in earlier surveys [10]. Smoking is considered a threat to oral and systemic public health [4,35]. Dentists and oral health care professionals play a major role promoting the cessation of smoking among their patients [36]. In the present investigation, the prevalence of smoking was 5.36% in preclinical and 8.62% in clinical students. Both groups showed much lower percentages of smoking than the general German population (28%) [37] and the recorded rates of cigarette smoking among German medical students (16%) [38]. Although the higher prevalence of smoking among clinical students is in agreement with previous studies, the result of the current survey displayed a mean smoking rate of dental students in Germany (11.30%) lower than in Bangladesh (22%), Holland (24%) Norway (24%), Greece (47%), Serbia (43%), Hungary (34%), France (33%), Italy (33%) and Turkey (26%). Furthermore, the numbers of daily smokers of 10 or more cigarettes per day among preclinical students (1.79%) and their clinical colleagues (5.17%) seem comparable to the mean rate reported among medical students in Germany (3%) [38]. Similarly, the frequency and length of smoking reported in the current study show lower rates compared to other countries [4].

Observing all items of the current study and the HU-DBI score of both participating groups, preclinical and clinical dental students in Germany reflected overall better oral health attitudes and behaviour than their colleagues in European, Middle-Eastern and Asian countries [4,10,11,17,18,31]. Indeed, Germany’s oral health system has proven to be one of the most efficient worldwide [39]. Nevertheless, the preclinical students presented a slightly higher, yet statistically insignificant, HU-DBI mean score than the clinical participants. This discrepancy with multiple studies performed worldwide [4,10,11,12,13,14,15,16,17] suggests an insignificant effect of the students’ preclinical–clinical transition and their required awareness as role models or educators to their patients. Furthermore, it might indicate possible difficulties during the progressive stages of this transition [7]. One of the factors that might lead to the observed decline in oral health attitudes and behaviour in the clinical group might be the increased stress during the clinical semesters, particularly during the phase of preclinical–clinical transition due to performance pressure and clinical requirements [7,40,41]. With increased levels of stress, students tend to neglect their oral health care leading to adverse effects on their oral and dental health [42]. This perceived stress might even show more pronounced effects on students living away from home in both of the study groups. Indeed, between getting used to classes, making new friends, settling into the dorms, as well as other situations and anxieties facing students living away from home along with simultaneous routine and diet changes, students might neglect their oral health [43,44]. Nevertheless, while this might affect the results of all students living away from home, it shows very slight differences between preclinical and clinical students in the current survey. Furthermore, the higher smoking rate of clinical students appears to be a possible factor affecting their periodontal health as observed in items 15 and 6 of the survey [45] and also correlates to high levels of academic stress during the clinical semesters [46]. Previous studies also noted a decreased self-care of medical students during the transition to the clinical stage of their education in spite of their increasing knowledge about health behaviour [47]. One of the important factors that should also be considered due to its role in the development of students’ health behaviour is the design and content of the university curricula [48,49,50]. In Germany, the vast majority of medical and dental schools are state- and tax-funded. They incorporate a standardized curriculum that concludes with the award of a medical or dental degree after successful accomplishment of all state board exams [5,51]. The curriculum defining the minimum requirements, number of classes and examination guidelines is designed by federal officials and successively written into law [5]. It intends to ensure that all students in German universities receive the same level of education and can provide equal patient care regardless of the location of their medical or dental training [5,51]. However, the universities have the freedom to implement these requirements in a design and order as they see suitable, on condition that they are following the legal guidelines [5,51]. Concerning the mentioned influence of curricula on the oral health manners and development of dental students, certain strategies might be needed in Germany to achieve the expected outcome of preclinical–clinical student transition. Curricular reviews and changes in dental schools to completely integrate behavioural and social sciences into the dental curricula besides biomedical knowledge could help students become holistic and patient-centred practitioners [50]. It also provides students with practical understanding of how to manage educational work-based stress and performance anxiety [50]. Furthermore, a curricular reformative approach looking to provide a correct and healthy transition from the preclinical to the clinical phase could likewise help the students become more responsible for their oral health as patient educators and for overall patient wellbeing [7]. As stated by participants of previous investigations, the difficult transition from pre-clinical to clinical training is a result of various and simultaneous educational variables, such as curricular design, content differences and organisational problems [7]. An early and gradual contact with the clinical environment, as well as integrative teaching between the preclinical and clinical training, could be suggested as a possible solution to improve the learning outcomes of the transitional stage to the clinical semesters [7,52].

### Limitations of the Study

The following limitations have to be acknowledged: First of all, as this is a cross-sectional study, any changes in HU-DBI scores cannot be attributed completely to the curricular level. Analysing co-variables such as gender, age, income and socio-environmental conditions could play a major role influencing the results of the survey. Ideally, sociodemographic analysis should be added to the survey with an evaluation of the same students in preclinical and clinical stages with additional clinical oral health assessment. Moreover, the present study examined a relatively small sample of students, mostly from the University of Kiel. The outcomes obtained may not be representative for other German universities or generalizable on all dental students in Germany. However, it could be important to give an indication of general oral health attitudes and behaviour of German dental students due to the equality of dental education in Germany as mentioned above. Furthermore, it is possible that students responding to the questionnaire reported better oral health attitudes and behaviour than they actually had, as dental students are familiar with correct dental health practices. In addition, evaluating simple items of the HU-DBI as the frequency of toothbrushing does not deliver the full picture concerning the overall quality of oral hygiene and might need clinical assessment.

## 5. Conclusions

Preclinical dental students of German universities showed a marginally higher HU-DBI score of oral health attitudes and behaviour than clinical students with statistically insignificant differences. This might reveal a non-effective transition of the students from the preclinical to the clinical stage to fulfil their role as oral health educators to their patients. A curricular review and reform are recommended to achieve the expected oral health manners from dental students and allow a successful preclinical–clinical transition. Further studies complemented by clinical assessment might be needed to explore the exact status of oral health in both groups and for a deeper investigation of their oral health attitudes and behaviour.

## Figures and Tables

**Table 1:** The modified English version of the Hiroshima University-Dental Behaviour Inventory (HU-DBI) survey in this study. Answers favouring correct oral health attitudes and behaviour were marked with (C).

**Item 1**	I live with My Family Now	Agree	Disagree
**Item 2**	I had been to a dentist office before	Agree (C)	Disagree
**Item 3**	I do not go to the dentist unless I feel pain	Agree	Disagree (C)
**Item 4**	I brush my teeth at least twice a day	Agree (C)	Disagree
**Item 5**	I brush my teeth after every meal	Agree (C)	Disagree
**Item 6**	My gums bleed when I brush my teeth	Agree	Disagree (C)
**Item 7**	I have been taught a professional brushing technique and I use it	Agree (C)	Disagree
**Item 8**	I use a toothbrush with hard bristles	Agree	Disagree (C)
**Item 9**	I brush each of my teeth carefully	Agree (C)	Disagree
**Item10**	I think my teeth are getting worse despite my daily brushing	Agree	Disagree (C)
**Item 11**	I use dental floss regularly	Agree (C)	Disagree
**Item 12**	I use a mouthwash regularly	Agree (C)	Disagree
**Item 13**	I worry about having bad breath	Agree (C)	Disagree
**Item 14**	I think I can clean my teeth without toothpaste	Agree	Disagree (C)
**Item 15**	I am bothered by the colour of my gums	Agree	Disagree (C)
**Item 16**	I worry about the colour of my teeth	Agree (C)	Disagree
**Item 17**	I am a smoker	Agree	Disagree (C)
**Item 18**	I smoke 10 or more cigarettes a day	Agree	Disagree (C)
**Item 19**	I have been smoking for a year or more	Agree	Disagree (C)
**Item 20**	I like snacking on sweets during the day	Agree	Disagree (C)

**Table 2:** Distribution of participating students by academic semester and gender.

Academic Semester	Total Number of Students (%)	Female Students (%)	Male Students (%)
1	8 (4.65%)	7 (5.88%)	1 (1.88%)
2	8 (4.65%)	7 (5.88%)	1 (1.88%)
3	13 (7.55%)	9 (7.56%)	4 (7.54%)
4	6 (3.48%)	4 (3.36%)	2 (3.77%)
5	21 (12.20%)	16 (13.44%)	5 (9.43%)
6	8 (4.65%)	5 (4.20%)	3 (5.66%)
7	17 (9.88%)	12 (10.08%)	5 (9.43%)
8	30 (17.44%)	18 (15.12%)	12 (22.64%)
9	18 (10.46%)	10 (8.40%)	8 (15.09%)
10	34 (19.76%)	24 (20.16%)	10 (18.86%)
11	9 (5.23%)	7 (5.88%)	2 (3.77%)
Total Numbers	172 (100%)	119 (100%)	53 (100%)

**Table 3:** Distribution of participating students by university.

University	Student Participation Number (%)
Christian-Albrechts-University of Kiel	57 (33.13%)
Private University Witten/Herdecke	14 (8.13%)
University of Freiburg	10 (5.81%)
Charite University Medicine Berlin	9 (5.23%)
TU Dresden	9 (5.23%)
FAU Erlangen-Nürnberg	9 (5.23%)
University of Bonn	8 (4.65%)
University of Hamburg	7 (4.06%)
Johannes-Gutenberg-University Mainz	5 (2.90%)
Goethe University Frankfurt	4 (2.32%)
Justus-Liebig-University Gießen	4 (2.32%)
University of Münster	4 (2.32%)
University of Düsseldorf	3 (1.74%)
University of Greifswald	3 (1.74%)
Philipps-University Marburg	3 (1.74%)
RWTH Aachen	2 (1.16%)
Martin-Luther-University Halle-Wittenberg	2 (1.16%)
Heidelberg University	2 (1.16%)
Saarland University	2 (1.16%)
University of Cologne	2 (1.16%)
Ludwig Maximilian University of Munich	2 (1.16%)
Regensburg University	2 (1.16%)
Julius-Maximilian-University Würzburg	2 (1.16%)
Georg-August-University Göttingen	1 (0.58%)
Leibniz University Hannover	1 (0.58%)
Jena University	1 (0.58%)
Leipzig University	1 (0.58%)
University of Rostock	1 (0.58%)
University of Tübingen	1 (0.58%)
Ulm University	1 (0.58%)

**Table 4:** Percentages and analysis of “Agree” and “Disagree” responses to items of oral health attitudes (OHA) and oral health behaviour (OHB) comparing the preclinical and clinical semesters’ dental students.

Item Number and “Keyword”	Number of “Agree” Answers (%)		Number of “Disagree” Answers (%)		*p*-Value
	Preclinical Semesters	Clinical Semesters	Preclinical Semesters	Clinical Semesters	
1 “Living with family” (sociodemographic)	8 (14.29)	17 (14.66)	48 (85.71)	99 (85.34)	0.57
2 “Visits to the dentist” (OHA)	56 (100.00)	115 (99.14)	0 (0.00)	1 (0.86)	0.67
3 “Visiting the dentist only when in pain” (OHA)	2 (3.57)	8 (6.90)	54 (96.43)	108 (93.10)	0.31
4 “Toothbrushing twice a day” (OHB)	49 (87.50)	107 (92.24)	7 (12.50)	9 (7.76)	0.23
5 “Brushing after every meal” (OHB)	1 (1.79)	8 (6.90)	55 (98.21)	108 (93.10)	0.14
6 “Bleeding gums when brushing” (OHB)	0 (0.00)	1 (0.86)	56 (100.00)	115 (99.14)	0.67
7 “Professional brushing technique” (OHB)	49 (87.50)	89 (76.72)	7 (12.50)	27 (23.28)	0.07
8 “Toothbrush with hard bristles” (OHB)	7 (12.50)	21 (18.10)	49 (87.50)	95 (81.90)	0.24
9 “Brushing each tooth” (OHB)	49 (87.50)	97 (83.62)	7 (12.50)	19 (16.38)	0.34
10 “Teeth get worse despite of brushing” (OHB)	9 (16.07)	18 (15.52)	47 (83.93)	98 (84.48)	0.54
11 “Regular dental floss” (OHB)	39 (69.64)	79 (68.10)	17 (30.36)	37 (31.90)	0.49
12 “Regular mouth wash” (OHB)	20 (35.71)	38 (32.76)	36 (64.29)	78 (67.24)	0.41
13 “Worrying about bad breath” (OHA)	17 (30.36)	30 (25.86)	39 (69.64)	86 (74.14)	0.33
14 “Tooth cleaning without toothpaste” (OHA)	1 (1.79)	11 (9.48)	55 (98.21)	105 (90.52)	* 0.05
15 “Bothered by the colour of gums” (OHB)	1 (1.79)	5 (4.31)	55 (98.21)	111 (95.69)	0.36
16 “Worrying about the colour of teeth” (OHA)	8 (14.29)	14 (12.07)	48 (85.71)	102 (87.93)	0.42
17 “Smoker” (OHB)	3 (5.36)	10 (8.62)	53 (94.64)	106 (91.38)	0.34
18 “10 or more cigarettes a day” (OHB)	1 (1.79)	6 (5.17)	55 (98.21)	110 (94.83)	0.27
19 “Smoking for a year or more” (OHB)	3 (5.36)	10 (8.62)	53 (94.64)	106 (91.38)	0.34
20 “Snacking on sweets during day” (OHB)	27 (48.21)	56 (48.28)	29 (51.79)	60 (51.72)	0.56

Chi-squared test, statistical significance marked with asterisk, * *p* < 0.05.

**Table 5:** HU-DBI Scores of preclinical/clinical and male/female dental students.

**HU-DBI Score**	**Preclinical Students**	**Clinical Students**	***p*** **-Value**
	14.62 ± 0.42	14.31 ± 0.43	0.170
	**Male Students**	**Female Students**	***p*** **-Value**
	13.33 ± 0.44	14.64 ± 0.42	* 0.001

Wilcoxon signed-rank test, statistical significance marked with asterisk, * *p* < 0.05.
